# The Effects of Uniquely-Processed Titanium on Balance and Walking Performance in Healthy Older Adults

**DOI:** 10.3390/jfb9020039

**Published:** 2018-06-08

**Authors:** Melissa J. Black, Adam A. Lucero, Philip W. Fink, Lee Stoner, Sarah P. Shultz, Sally D. Lark, David S. Rowlands

**Affiliations:** 1 School of Sport, Exercise and Nutrition, Massey University, Wellington 6021, New Zealand; M.Black@massey.ac.nz (M.J.B.); adamluco1@gmail.com (A.A.L.); P.Fink@massey.ac.nz (P.W.F.); S.P.Shultz@massey.ac.nz (S.P.S.); S.Lark@massey.ac.nz (S.D.L.); 2 Department of Exercise and Sport Science, University of North Carolina, Chapel Hill, NC 27514, USA; dr.l.stoner@gmail.com

**Keywords:** titanium, recovery, balance, blood flow, perfusion, stretch reflex, range of motion, walking

## Abstract

The increased risk of falls associated with advancing age has increased demand for methods to improve balance and mobility. The primary purpose of the study was to determine whether wearing Aqua Titan-treated stockings could improve balance and walking performance in an older population; secondary was to elucidate the mechanisms. In a randomized, double-blind crossover, 16 healthy older adults (age, 67.9 ± 4.2 years; BMI, 24.8 ± 3.1 kg/m^2^) performed two 4-day trials composed of baseline measures and fatiguing exercise on Day 1, with recovery measures at 14, 38 and 62 h post-exercise, wearing Aqua Titan and control stockings. Balance, walking performance, triceps surae stretch reflex, ankle range of motion and gastrocnemius muscle microvascular perfusion, blood flow and oxygen consumption were measured at baseline and during recovery. Aqua Titan had no effect on the microvascular parameters, but increased total ankle range of motion at 38 h (2.4°; 95% CI ± 1.8°) and 62 h (2.7°; ±1.7°), contributed to by increases in dorsiflexion and plantar flexion. There was decreasing persistence in the medial-lateral center of pressure movement at 38 h (q = 0, −0.0635 ± 0.0455), compared to control stockings. Aqua Titan garments hold potential for improving balance and mobility in older adults in the days following a bout of fatiguing exercise. The proposed mechanisms associated with enhanced sensory feedback require further exploration.

## 1. Introduction

The latter years of human life are associated with an increased risk of falls; a consequence of senescence of skeletal muscle, related vasculature and impaired sensory ability [1,2]. Subsequently, there is a demand for products designed to help reduce fall-related injuries among older adults. Titanium metal has been well established as a valuable biomedical tool in a variety of areas including, but not limited to, dental materials [3] and orthopedic implants [4]. Titanium’s relative abundance, bioavailability and high strength-to-weight ratio all contribute to its popularity in past and present biomedical research [5]. Aqua Titan^®^ is a novel product consisting of picometer to micrometer sizes of titanium, melted by the combustion gas of hydrogen and oxygen in high-pressure water, then further dissolved in high-function water [6]. More recently, the development of Aqua Titan has widened the scope of titanium use, through embedding and bonding nanoparticles of titanium into fabric via utilization as a dye. Of relevance to the aging population, the physiological properties of Aqua Titan may help to minimize the effects of age-related degeneration in muscles and joints, subsequently lessening the chance of falls and improving quality of life. 

Aqua Titan garments and tapes have been investigated in a range of environments; from demonstrations in vitro of alterations to the synaptic plasticity of neurons in acute hippocampal slices from mice [7], to in vivo evidence suggesting reduced psychological and physiological stress [8] and enhanced musculoskeletal recovery from strenuous activity [9,10,11]. Of particular importance to the current study are the reports of superior recovery from high-intensity exercise whilst wearing Aqua Titan garments or tape. The enhanced recovery has been characterized by an improvement in joint range of motion (ROM) [9,11], restoration of Achilles tendon stiffness [9], enhanced short latency stretch reflex response [9] and a lower metabolic cost of running [10], relative to control garments. Collectively, these findings suggest Aqua Titan applied to the skin has positive effects on restoration of musculoskeletal function, more specifically at the muscle-tendon complex, with potential for enhancement in neuromuscular functioning and fine-motor control. Whilst the mechanisms behind these improvements remain speculative and require further investigation, the observed improvements may have particular relevance amongst an older population, where poor muscle quality contributes to the increased risk of falls. Exploring the effects of Aqua Titan during the recovery from a bout of strenuous exercise in older adults may help elucidate possible mechanisms, as well as enabling the investigation of Aqua Titan-induced restoration of fatigued/damaged aged skeletal muscle.

The primary purpose of the current study was to investigate an older cohort and to determine whether Aqua Titan, applied in the form of a stocking, improves balance and walking performance during the recovery period after a bout of strenuous exercise. The secondary purpose was to elucidate the potential explanatory mechanisms, through measurement of muscle microvascular blood flow, perfusion and oxygen consumption, and mechanisms associated with mobility including the triceps surae short latency stretch reflex response and ankle joint range of motion (ROM). We hypothesized that Aqua Titan would increase microvascular blood flow to the gastrocnemius muscle during the recovery period, maintain normal rested baseline muscle-tendon function and improve balance and walking performance, relative to a control.

## 2. Materials and Methods 

### 2.1. Participants

Sixteen healthy and recreationally-active 60–80 years old participants volunteered to participate in the study. Characteristics of the participants are displayed in Table 1. Potential participants completed a health screening questionnaire and were interviewed prior to the onset of the study to determine whether they met the inclusion/exclusion criteria. Potential participants were excluded if they were not recreationally active (self-reported participation in >150 min of moderate-intensity exercise, or >75 min of vigorous-intensity exercise per week), unable to safely walk on a treadmill at a speed of at least 4 km·h^−1^ for 35 min, had complaints of Achilles or plantar-flexor tendinopathy, joint disease, severe lower limb skin disorders, changes in nerve sensation, critical limb ischemia, severe kidney or liver disease, unstable angina and were using analgesic medication, compression stockings or a walking aid. Participants were also excluded if adipose tissue thickness was >10 mm at the site of the near infrared spectroscopy (NIRS) measurements. All participants were informed about the potential risks and provided written consent for their participation in the study. The study was conducted in accordance with the Declaration of Helsinki, and the protocol was approved by the Massey University Ethics Committee (16/49).

### 2.2. Experimental Design

**Figure 1:**
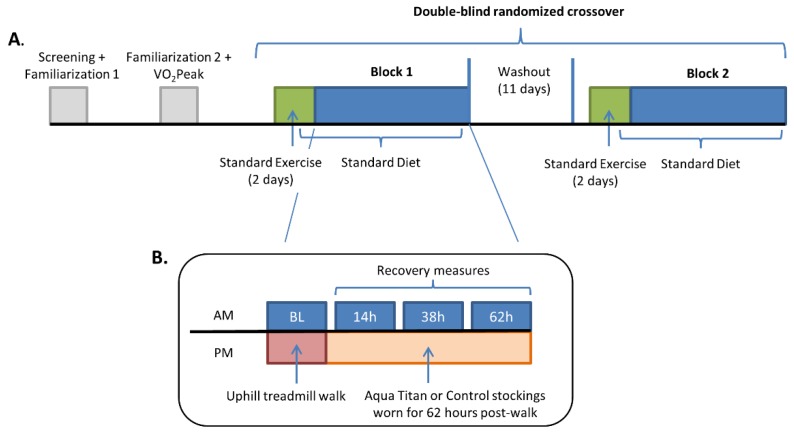
Experimental design: (**A**) four-week testing period, consisting of two initial familiarization/screening sessions including a VO_2_Peak test and the two crossover blocks; (**B**) detail of the four-day testing block. BL = Baseline.

Each participant completed two experimental blocks; one where Aqua Titan stockings were worn, and the other where the control stockings were worn (Figure 1). The Aqua Titan and control stockings were assigned a number (1 or 2) by a member outside of the research team, allowing allocation of the order of Aqua Titan or control stockings to be done in a randomized, double-blind manner using a random number generator function in Excel. To prevent any mixing of the treatments, the stockings were stored in separate rooms and identified by differing colors stitched into the inside hem. The stockings were tight fitting, but not compressive, and custom made by the Aqua Titan manufacturers Phiten Co. Ltd. (Kyoto, Japan), using a method previously described [6].

### 2.3. Preliminary Sessions

Participants’ weight and height were measured on their first visit to the laboratory using electronic scales (model X-3AM; Hiweigh, Shanghai, China) and a standard laboratory stadiometer (Surgical and Medical Supplies Pty. Ltd., Rose Park, Australia). Ultrasound (Terason, United Medical Instruments Inc., San Jose, CA, USA) measurements were taken on the right calf muscle to quantify adipose tissue thickness and depth from skin to medial gastrocnemius (GM) and to determine the appropriate site for the NIRS blood flow measures. Isometric plantar flexion maximal voluntary contraction (MVC) was obtained on the right leg through the maximum of three trials using an isokinetic dynamometer (Biodex Medical Systems, System 3, Shirley, NY, USA). The participant was seated on the dynamometer and reclined to 70°, eliciting a 110° hip angle with his/her leg extended and foot strapped into a foot plate, positioned at 10° of plantar flexion. The dynamometer was adjusted for each participant to align the lateral malleoli of the ankle with the axis of rotation of the dynamometer. The dynamometer settings for each participant was recorded and replicated for each session.

Peak oxygen uptake of each participant was measured using a treadmill-based incremental walking protocol combined with expired air gas analysis (Sensormedics Vmax, San Diego, CA, USA). The walking protocol was based on the Northbridge standardized exponential exercise protocol (STEEP) [12], in which the speed or the incline is increased every minute, until volitional fatigue. Heart rate was monitored throughout the test. During their first two visits to the laboratory, the participants were introduced to all of the measurements involved in the study to minimize the impact of any learning effect.

### 2.4. Main Trial Procedures

Prior to beginning their first testing block, participants were required to record an exercise diary for the two days before their first session and a diet diary from 24 h prior to their first session through to the last day of their first block. To help eliminate additional variation, participants were instructed to replicate their exercise and diet prior to and during their second testing block. Participants were asked to refrain from avoidable physical activity throughout each block. All sessions during the four-day blocks (with the exception of the afternoon/evening uphill walk) had the requirement of an overnight fast with only the consumption of water allowed. Participants were instructed to refrain from caffeine intake for at least 12 h prior to each session and alcohol consumption 24 h prior to each session.

Baseline measures of ankle ROM, balance, Achilles tendon short latency stretch reflex response (SLRx), muscle microvascular perfusion (Perf), muscle microvascular blood flow (mBF) and muscle oxygen consumption (mVO_2_) were collected in the morning of the participants’ Day 1 of their testing block. Participants returned to the laboratory approximately 10 h after the start of their previous visit to complete a treadmill-based walking protocol designed to fatigue the GM. All participants completed a 35-min treadmill walk at 4.4 ± 0.6 km·h^−1^. The walk consisted of a 5-min warm-up period at a 0% gradient, followed by 4 × 5-m uphill sections at a 10% gradient, separated by 2-min recovery walking periods at a 0% gradient and a 4-min cool-down. Participants were instructed to walk on their forefoot during the uphill sections in order to maximize GM activation. Participants reported exertion levels as ‘hard’ on the uphill walking sections. Within 10 min of completing the treadmill walk, the participants were provided with the stockings, which they wore for the remainder of the testing block, excluding whilst in bed and during any water-based activities. The stockings were worn throughout all testing procedures; a hole was cut in the right stocking at the site of the GM to allow for short latency stretch reflex response and NIRS measurements. A new pair of stockings was provided to the participants each day.

Participants reported to the laboratory for the next three subsequent days to complete the 14-, 38- and 62-h recovery measures. All three of these visits were at the same time of day as their baseline measurements and consisted of ROM, SLRx, balance, Perf, mBF and mVO_2_, with the addition of the submaximal whole body oxygen consumption (SubmaxVO_2_) and walking performance measures on the final testing day of each block.

#### 2.4.1. Ankle Range of Motion

Ankle dorsiflexion and plantar flexion were measured on the right leg using a goniometer (Baseline Instruments, Auckland, New Zealand). Participants were seated on a massage table with their leg extended and heel just over the edge. They were instructed to keep their knee fully extended throughout maximal dorsiflexion and plantar flexion. Three measures of each were performed each session, and the results averaged.

#### 2.4.2. Balance

Participants positioned themselves on the plantar pressure plate (RSscan International FootScan^®^, RSscan International NV, Paal, Belgium), in a bilateral stance with their feet together and hands by their sides. Participants were instructed to look straight ahead and stand as still as possible prior to closing their eyes. All trials were completed with eyes closed in order to remove the visual stimulus and place emphasis on the vestibular and somatosensory systems. The pressure plate data were collected at a sampling rate of 100 Hz for 10 s, with a 10-s lead in and a 10-s lead out. Displacement of the x (medial-lateral) coordinates of the center of pressure (COP) were analyzed using multifractal detrended fluctuation analysis (MFDFA) [13]. MFDFA divides the time series into eight segments, ranging in length from 8 data points to 1000 data points, equally spaced on a logarithmic scale. For each segment, a detrended root mean square (RMS) value was calculated. The RMS was weighted by a parameter q, which varied from −3–5, where small q values weight small fluctuations more heavily and large q values weight large fluctuations more heavily. Temporal correlations within the time series were measured using the slope of the RMS, weighted by different q values vs. sample length (Hq). Hq values greater than 0.5 indicated that the time series was persistent, where increases from one time step to another would be more likely to be followed by further increases and decreases more likely to be followed by further decreases. Hq values of less than 0.5 indicated an anti-persistent time series, where increases would be more likely to be followed by decreases and vice versa. Thus, Hq provides a way of analyzing whether motions of the COP would be corrected (anti-persistent) or continue to drift (persistent). Ability to balance can then be inferred, where smaller Hq values (decreasing persistence) indicate better correction for fluctuations.

#### 2.4.3. Triceps Surae Short Latency Stretch Reflex Response

SLRx was determined through surface electromyographic (sEMG) activity of the GM in response to a force applied to the Achilles tendon via a tendon hammer (ADIntruments, Bella Vista, Australia). sEMG activity was recorded from the right GM by applying two bipolar surface electrodes (Ambu^®^ Blue Sensor N, Ambu A/S, Ballerup, Denmark) with a 10 mm diameter and a 20-mm inter-electrode distance at the site of the NIRS measurements. Prior to electrode placement, the area was shaved, lightly abraded and cleaned with 70% isopropyl alcohol wipes (Webcol, Covidien, Walpole, MA, USA). Participants were positioned on their hands and knees on the massage table with their toes just over the edge. Participants were instructed to place weight through their arms and keep their legs as relaxed as possible. The Achilles tendon was gently tapped with the instrumented tendon hammer, whilst the sEMG and hammer signal were collected at 1000 Hz using an ADI power lab system (Powerlab 4/25, ADInstruments, Australia). The tendon hammer contained a piezo-electric sensor within the head to provide a momentary pulse when a surface was struck with the hammer. The sEMG signal was amplified (BioAmp, ADInstruments, Australia) and integrated into LabChart software (LabChart v8.1.5, ADInstruments, Australia). Markers were manually added into the LabChart software at the first point of contact of the tendon hammer and at the initial deflection of electrical activity of the GM from baseline. SLRx was calculated from the time difference between the two markers. Five measures were performed each trial and the results averaged.

#### 2.4.4. Muscle Microvascular Perfusion, Blood Flow and Oxygen Consumption

Participants were seated on the Biodex dynamometer based on their settings established in the familiarization. The NIRS device was secured via double-sided tape and hook and loop straps and applied to the muscle belly of the GM at the site established via ultrasound measures. A black cloth was placed over the NIRS probe to eliminate all ambient light, which was held in place via a bandage lightly wrapped around the probe and the participants’ leg so as not to constrict blood flow. After being seated and still for 10 min, baseline mBF and mVO_2_ measurements were taken via three venous occlusions (VO) and two arterial occlusions (AO), respectively. Occlusion durations were 10 s for VO and 15 and 30 s for the first and second AO, with all five occlusions separated by 90 s. Resting perfusion was measured after the participants had been still for at least 10 min, but prior to the first occlusion. Upon completion of resting measurements, participants performed plantar flexion exercise on the isokinetic dynamometer at a set rhythm of one plantar flexion movement every three seconds for 10% and then 20% of MVC. Participants were instructed to push through the ball of their foot to move the footplate in a downwards motion, where at the bottom of their ROM, they would relax and allow the dynamometer to return their foot to the starting position (10° of plantar flexion). At 10% and 20% MVC, participants exercised continuously for five minutes to establish steady state blood flow prior to the first occlusion. Exercise perfusion was calculated from the average resting total hemoglobin (t[Hb]) signal between the final 10 plantar flexion movements, prior to the first occlusion. After five minutes of exercise, participants were notified of their final three plantar flexion movements, where immediately after the final movement, they completely relaxed and the cuff was rapidly inflated for an eight-second VO. Upon cessation of the VO, participants immediately continued the plantar flexion exercise at the same rhythm for another 80 s prior to another occlusion. Three VOs and two AOs were completed during the 10% and 20% exercise protocols. The data for the three VOs and two AOs were averaged to give a single value for each, at rest and during the two exercise intensities. A detailed description of NIRS and occlusion processes and calculations of muscle perfusion, blood flow and oxygen consumption are presented in Appendix A. 

#### 2.4.5. Submaximal Oxygen Consumption and Walking Performance

SubmaxVO_2_ consisted of a three-stage incremental walking protocol, based on the first three stages of the modified Bruce protocol [14], combined with expired air gas analysis. The walking test was completed straight after the SubmaxVO_2_ and used the same protocol as the determination of peak oxygen consumption, described in Section 4.3. Participants were blinded to time and heart rate throughout the test, where they completed as much of the protocol as possible. Time and heart rate at volitional exhaustion were recorded.

### 2.5. Statistical Analysis

Data were analyzed using a mixed model (SAS, Cary, NC, USA) structured specifically for post-pre crossover designs described by Kenward and Roger [15]. Briefly, the fixed effect model was the period number × type interaction and the group × type interaction, where type was either baseline or post intervention sample. Random effects were the type with the subject term being the participant ID; the random effects model type was unstructured. A repeated effect was included as the type with the subject term being the participant × period number interaction. Estimates were the mean and 95% confidence interval. Inference was by the method of magnitude-based inference. In brief, magnitude-based inference quantifies the probability of a mean effect outcome greater than the smallest defined meaningful or standardized effect size threshold, which in the current case is the smallest Glass’s d effect size (difference in (Aqua Titan post treatment − control post treatment)/baseline); this compares to testing against the null (smallest effect = zero). Meanwhile, the probability of an inverse smallest meaningful effect is also derived. Probabilities are drawn directly from the *t*-distribution. Accordingly, the probability that a contrast was at least greater than the smallest standardized difference (0.2 × baseline SD) was: 25–75% possible, 75–95% likely, 95–99.5% very likely, >99.5% almost certain. In the case where the majority (>50%) of the CI were between the thresholds for positive and negative substantiveness, the effect was qualified as trivial (negligible) with the respective probabilities as above. The terms increase, trivial (negligible) and decrease refer to the most likely directional outcome, relative to the smallest effect threshold. The terms unclear and inconclusive refer to outcomes where the likelihood of both increase and decrease exceeded 5%. A more detailed account of magnitude-based inference has been described elsewhere [16,17].

## 3. Results

### 3.1. Effects of Aqua Titan-Treated Stockings on Balance

**Figure 2:**
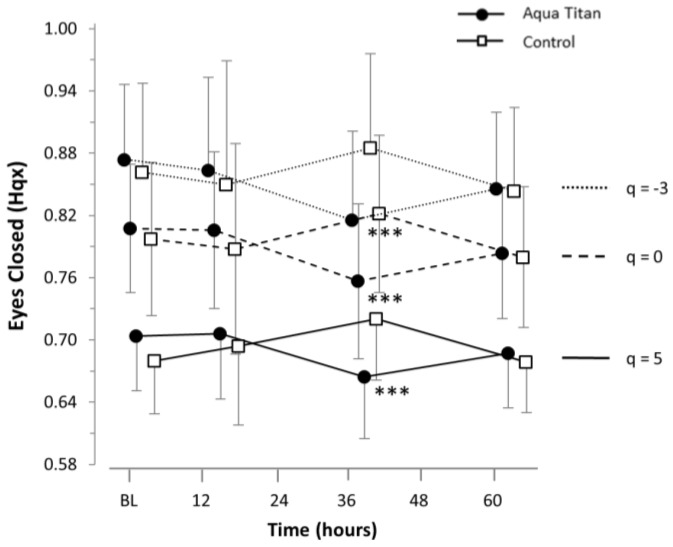
Effect of Aqua Titan-treated stockings worn during the 62-h recovery period after an uphill walk on medial-lateral center of pressure (COP) movement in a bilateral stance with eyes closed, relative to the pre-walk baseline (BL). Data are the mean ± SD. The threshold for the smallest substantial meaningful change on the balance function of Hq was set at 0.2 × SD [18]. The number of symbols denotes the likelihood of a difference between Aqua Titan and Control groups, where: * = possible; ** = likely; *** = very likely; **** = almost certain.

Medial-lateral movement of COP indicated decreasing persistence whilst wearing Aqua Titan-treated stockings at 38 h into recovery, relative to the control (Figure 2, Table A1). Hq values were very likely lower across all three q indices measured, indicating that the reduced persistence was at all ranges of fluctuation size.

### 3.2. Effects of Aqua Titan-Treated Stockings on Muscle Microvascular Perfusion, Blood Flow and Oxygen Consumption

**Figure 3:**
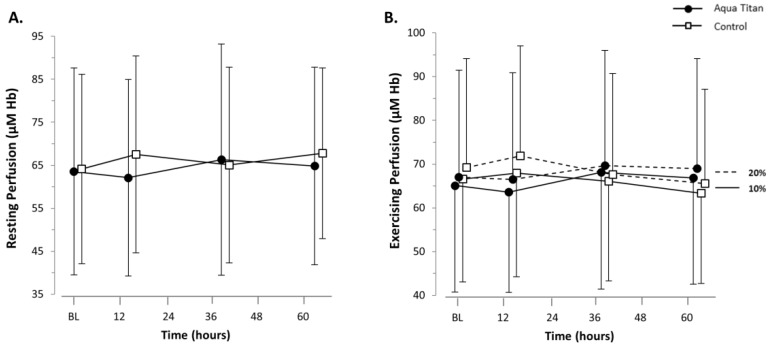
Effect of Aqua Titan-treated stockings worn during the 62-h recovery period after an uphill walk on (**A**) muscle microvascular perfusion at rest and (**B**) during 10% and 20% plantar flexion exercise, relative to the pre-walk baseline (BL). Mean data ± SD are presented. There were no substantial effects of treatment.

**Figure 4:**
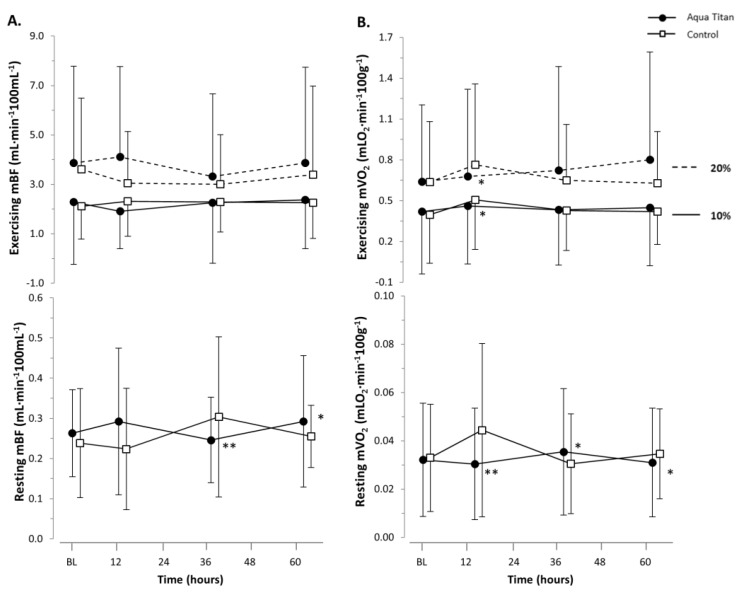
Effect of Aqua Titan-treated stockings worn during the 62-h recovery period after an uphill walk on (**A**) muscle microvascular blood flow (mBF) and (**B**) oxygen consumption (mVO_2_) at rest and during 10% and 20% plantar flexion exercise, relative to the pre-walk baseline (BL). Data are the mean ± SD. The threshold for the smallest substantial meaningful change on mBF and mVO_2_ was set at 0.2 × SD [18]. The number of symbols denotes the likelihood of a difference between Aqua Titan and control groups, where: * = possible; ** = likely; *** = very likely; **** = almost certain.

While the Aqua Titan effect sizes, relative to the smallest standardized difference, were not substantial (trivial), physiological patterns were evident. Muscle microvascular resting perfusion was lower at 14 h into recovery. During plantar flexion exercise, Aqua Titan-treated stockings increased perfusion by around 3 µM Hb at 38 and 62 h into recovery (Figure 3, Table 2). There were no clear effects of Aqua Titan on muscle microvascular blood flow or oxygen consumption, as the data did not reach a level of statistical likelihood where we are able to draw firm conclusions (Figure 4, Table 3).

### 3.3. Effects of Aqua Titan-Treated Stockings on Ankle Range of Motion

**Figure 5:**
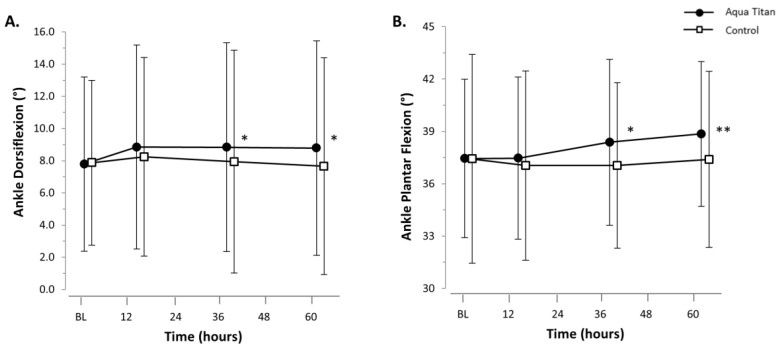
Effect of Aqua Titan-treated stockings worn during the 62-h recovery period after an uphill walk on ankle (**A**) dorsiflexion and (**B**) plantar flexion, relative to the pre-walk baseline (BL). Data are the raw means and SDs. The threshold for the smallest substantial meaningful change on dorsiflexion and plantar flexion was set at 0.2 × SD [18]. The number of symbols denotes the likelihood of a difference between the Aqua Titan and control groups, where: * = possible; ** = likely; *** = very likely; **** = almost certain.

Aqua Titan-treated stockings led to a possible increase in dorsiflexion ROM at 38 h (1.1°; 95% CI ± 1.2°) and 62 h (1.2° ± 1.5°) into recovery, relative to the control stockings. There was a possible increase in plantar flexion ROM at 38 h (1.3° ± 0.9°) and a likely increase at 62 h (1.4° ± 1.1°) into recovery with Aqua Titan, relative to the control (Figure 5, Table A2). Overall, total ankle ROM was likely increased at 38 h (2.4° ± 1.8°) and very likely increased at 62 h (2.7° ± 1.7°), equating to a 6% improvement with the application of Aqua Titan (Table A2). 

### 3.4. Effects of Aqua Titan-Treated Stockings on Submaximal Whole Body Oxygen Consumption and Maximal Walking Performance

**Figure 6:**
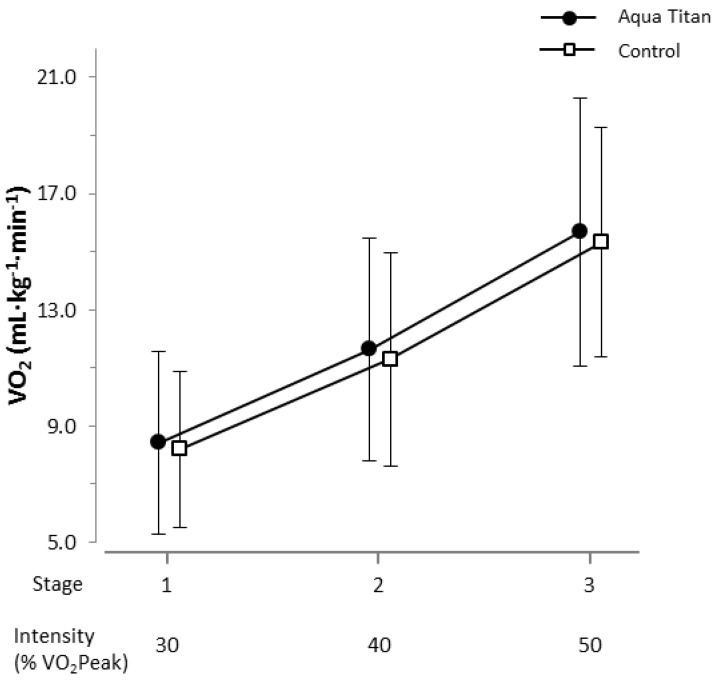
Effect of Aqua Titan-treated stockings on submaximal whole body relative oxygen consumption. Data are the raw means and SDs. The threshold for the smallest substantial meaningful change on relative oxygen consumption was set at 0.2 × SD [18]. There were no substantial effects of treatment.

Aqua Titan-treated stockings had no clear effect on submaximal whole body oxygen consumption assessed at 30%, 40% and 50% of VO_2_ Peak, relative to the control (Figure 6). The effect of Aqua Titan-treated stockings on walk time to exhaustion was unclear, relative to control (539.6 ± 87.9 s vs. 535.4 ± 95.8 s, respectively).

## 4. Discussion

The primary purpose of the current study was to investigate the effect of Aqua Titan on balance and walking performance among healthy older adults. The secondary purpose was to investigate possible explanatory mechanisms through measurements of muscle microvascular blood flow, oxygen consumption and perfusion, short latency stretch reflex response and joint ROM throughout a 62-h recovery period after an uphill walk. Aqua Titan-treated stockings improved balance at 38 h, but did not have any effect on walking performance. There were no clear effects of Aqua Titan on muscle microvascular blood flow or oxygen consumption, but there was some evidence for increased muscle microvascular perfusion during plantar flexion exercise. Aqua Titan-treated stockings improved ankle joint ROM from 38 h into recovery.

### 4.1. Aqua Titan-Treated Stockings Improved Balance, But Had No Effect on Walking Performance or Economy

There are clear associations with increasing age and declining neuromuscular control [19,20,21]. Even though the monosynaptic short latency stretch reflex exists at the lowest level of the nervous system’s postural control hierarchy [22], enhancement could lead to improvements in maintenance of balance through faster corrections to postural adjustments. The monosynaptic short latency stretch reflex response is dependent on a precise balance of excitatory and inhibitory information from neural receptors in the spinal cord to produce motor neuron excitability [23]. The muscle spindle provides essential information on muscle length and relays this to the spinal cord for integration and appropriate response via the α- and γ-motor neurons. When the muscle is in an unstretched state, action potentials are generated at a constant rate, whereas when stretched, the muscle spindle is activated and the action potential firing rate increases [24]. Unfortunately, we were unable to quantify the effect of Aqua Titan on the stretch reflex response in older adults, due to the inability to produce the ankle jerk response in a number of our participants, an issue that has been noted in previous literature [25]. Nevertheless, Hughes, et al. [9] observed an almost certain large reduction in short latency stretch reflex among their younger participants with Aqua Titan tape application, using the Achilles tendon tap method. The reduced reflex time suggests enhanced neuromuscular functioning via faster nerve conduction; although the mechanism behind this improvement remains untested. Increased action potential firing rate can lead to faster neural communication, but an action potential is an energetically expensive process; a sensory neuron expends between 30–75-times more energy during an action potential, compared to when in a state of rest [26]. An important aspect that impacts the energy cost of neural processing is the amount of noise (redundant information) amongst the signal production [27]. If the amount of noise involved in a sensory feedback system can be reduced, said system becomes more efficient [27]. The previous evidence by Korte [7] showing reduced action potential firing rates with the application of Aqua Titan material makes us hypothesize that there is an enhancement in neural signaling efficiency. Therefore, it may be this efficiency (rather than increased firing rate) that has led to past improvements in the short latency stretch reflex, and this may be a potential mechanism behind the enhanced balance observed in the current study. Completing the balance task without any visual input places more emphasis on the vestibular and somatosensory systems and infers that the observed improvement with Aqua Titan is due to enhanced neuromuscular feedback and/or coordination.

Interestingly, the improvement in balance was only noted at 38 h into recovery, but not at 14, or 62 h. There is the possibility that the neutral result at 62 h may be driven by neural acclimation, but similar fluctuations in Aqua Titan-induced benefits to recovery have also been observed in previous studies. Wadsworth, et al. [11] reported a reduction in peak run velocity on Day 3, but an improvement on Day 5 of recovery. In the same study, there were inconsistencies between recovery days for the ROM measures; some improvements being conclusive on one day, but trivial on another, or vice versa. If there are potential oscillations in the Aqua Titan effect on recovery, these will need to be investigated further to provide insight into the optimal time and duration of application.

Although we did not observe any improvement in walking performance or economy (contrary to previous research [10]), the extent of Aqua Titan coverage needs to be considered. Rowlands, et al. [10] employed whole body coverage of Aqua Titan using tight fitting garments and socks; however, only the lower legs were exposed to Aqua Titan in the current study. Whilst an Aqua Titan effect at the muscle-tendon complex was observed, the small extent of coverage may not have been enough to induce improvements in walking performance or efficiency. The association between Aqua Titan coverage and improvements in performance/efficiency needs to be investigated further.

### 4.2. Aqua Titan-Treated Stockings Had No Clear Effects on Muscle Microvascular Blood Flow, Perfusion or Oxygen Consumption

Following high-intensity fatiguing exercise, inflammation occurs as a result of damage to the skeletal muscle via mechanical and metabolic stresses [28]. Increased blood flow associated with inflammation could advance the delivery of substrates carried in the blood required for muscle repair and regeneration. An interesting outcome of the current study is the lack of evidence to suggest that there are any changes in muscle microvascular blood flow or oxygen consumption associated with the application of Aqua Titan-treated stockings. While muscle microvascular blood flow and oxygen consumption were unaffected, there was some evidence for increased muscle microvascular perfusion during plantar flexion exercise from 38 h into recovery. Greater perfusion would suggest enhanced dilation of the arterioles feeding the associated capillary beds; but because of the trivial effect size, combined with the lack of microvascular blood flow or oxygen consumption changes, the physiological relevance of the effect remains to be confirmed. Our current findings therefore challenge the idea that enhanced musculoskeletal recovery arises via Aqua Titan-induced microvascular blood flow changes [29].

### 4.3. Aqua Titan-Treated Stockings Increased Ankle Joint Range of Motion

The increases in dorsiflexion and plantar flexion ROM are consistent with findings from previous observations [9,11], reinforcing the proposal that Aqua Titan has an effect on the muscle-tendon complex. The exact cause behind the observed improvements has yet to be resolved; the original proposition of increased tendon compliance was debunked by Hughes, et al. [9], who reported tendon stiffness to be maintained following strenuous loading exercise, rather than reduced. Previous research associated with stretching-related improvements in joint ROM has focused on the mechanical and neuromuscular contributions, both of which have been well documented amongst the literature [30,31]. More recently, there has been increasing focus on the sensory aspect of muscle extensibility and its potential influence on joint movement. Through a comprehensive review of the literature associated with stretching, Weppler and Magnusson [32] concluded that increased muscle extensibility, often observed after stretching, is due to alterations in subject sensation (i.e., pain onset or tolerance), rather than mechanical changes. An in vitro investigation of Aqua Titan tape demonstrated impaired synaptic plasticity (long-term potentiation) in pyramidal neurons of the mouse hippocampus [7], which may lead to a reduction in pain sensation and memory [33]. Alongside being a potential explanation for the observed increase in joint ROM, impaired synaptic plasticity also provides an explanation of the anecdotal reports of analgesia with the application of Aqua Titan-treated tape and patches.

### 4.4. Considerations and Limitations

The participant population investigated in the current study introduced a large degree of between-subject variation across many of our outcomes. This issue was particularly noticeable when measuring the short latency stretch reflex response using the Achilles tendon tap method, such that some participants displayed a very clear response, whilst others had no obvious ankle jerk, or there was too much noise to confidently draw conclusions. Similarly to the muscle microvascular blood flow, perfusion, oxygen consumption and ROM measures, the large standard deviations illustrate the variation amongst the dataset with the given participants. Basal microvascular resting perfusion was a good example of this, with participant values ranging from 29–108 µM Hb. These are factors that need to be taken into consideration for future research; it may be more suitable to involve a narrower age range within the elderly population, e.g., 65–70 years or 70–75 years, or stratify to limit variation in the basal characteristics of the primary outcomes. This may help address some of the between-subject variation that may account for physiological sensitivity behind individual responses to treatment.

The laboratory equipment used for the balance measures must be noted as a potential limitation. The type of pressure plate available (and associated software) only allowed a maximum of 10 s of data collection at 100 Hz. While this was sufficient for the analysis, collection over a longer period of time (i.e., 30 s), and at a higher sampling rate, may have provided greater insight into some of the balance patterns and apparent improvements emerging.

## 5. Conclusions

The application of Aqua Titan-treated stockings worn during a 62-h recovery period following a strenuous uphill walk improved balance, but did not have any effect on walking performance in healthy 60–80-year-old adults. The explanatory variables we investigated concluded that the previously reported enhanced musculoskeletal recovery from hard exercise with the application of Aqua Titan was likely not due to microvascular changes, but rather muscle-tendon and neuromuscular changes proving as more likely mechanisms. Aqua Titan induced improvements in joint ROM that were consistent with previous findings and were likely due to changes in stretch or pain sensation rather than mechanical factors, possibly associated with the previously-reported alteration in the synaptic plasticity of nerve cells. Balance was improved at 38 h into recovery with the application of the Aqua Titan stockings; potentially a result of a more efficient sensory feedback system, enabling faster corrections to postural adjustments, while the neutral result at 62 h may be driven by neural acclimation to the Aqua Titan modulating effect. The newly-proposed mechanisms need to be investigated in more detail, as do the temporal effects of garment application to further explore the potential for materials containing Aqua Titan to improve mobility in older aged individuals.

## Figures and Tables

**Table 1:** Baseline characteristics for the 16 completed participants.

Characteristic	n = 16
Age (year)	67.9 ± 4.2
Gender (male, female)	8, 8
Height (m)	1.68 ± 0.11
Weight (kg)	70.1 ± 12.8
Body mass index (kg/m^2^)	24.8 ± 3.1
Adipose tissue thickness at NIRS site (mm)	4.7 ± 1.7
Peak oxygen uptake (mL·kg^−1^·min^−1^)	35.0 ± 10.4
All values are the mean ± SD	

**Table 2:** Statistical summary of the effect of Aqua Titan-treated stockings worn during the 62-h recovery period after an uphill walk on muscle microvascular perfusion at rest and during 10% and 20% plantar flexion exercise, relative to the pre-walk baseline.

Contrast ^a^	Change	Lower CI	Upper CI	*p*-Value	Likelihood (%) Decrease/Trivial/Increase	Qualitative ^b^
	**µM Hb**				**Resting Perfusion (µM Hb)**	
14 h–BL AqT–Control	−3.11	−7.44	1.22	0.141	28.6/71.4/0.03	Possibly trivial
38 h–BL AqT–Control	1.70	−2.06	5.47	0.337	0.08/91.2/8.75	Likely trivial
62 h–BL AqT–Control	0.722	−1.66	3.10	0.515	0.01/99.5/0.51	Very likely trivial
					**10% Exercise Perfusion (µM Hb)**	
14 h–BL AqT–Control	−2.02	−6.93	2.89	0.377	14.5/85.2/0.30	Likely trivial
38 h–BL AqT–Control	3.54	0.140	6.94	0.042	0.00/71.7/28.3	Possibly trivial
62 h–BL AqT–Control	3.07	−3.14	9.28	0.158	0.02/63.3/36.7	Possibly trivial
					**20% Exercise Perfusion (µM Hb)**	
14 h–BL AqT–Control	−1.83	−8.87	5.21	0.578	18.7/79.0/2.36	Likely trivial
38 h–BL AqT–Control	3.05	−0.668	6.77	0.097	0.00/81.0/19.0	Likely trivial
62 h–BL AqT–Control	3.88	−1.57	9.33	0.137	0.05/62.8/37.1	Possibly trivial

^a^ Data for each contrast are 14-h baseline, 38-h baseline and 62-h baseline. ^b^ The threshold for the smallest worthwhile change for a perfusion was 0.2 × SD [18]. The likelihood that a contrast was at least greater than the Cohen *d* threshold was: 25–75% possible; 75–95% likely; 95–99.5% very likely; >99.5% almost certain [18]. BL = baseline; AqT = Aqua Titan; CI = 95% confidence interval; Hb = hemoglobin.

**Table 3:** Statistical summary of the effect of Aqua Titan-treated stockings worn during the 62-h recovery period after an uphill walk on muscle microvascular blood flow and oxygen consumption at rest and during 10% and 20% plantar flexion exercise, relative to the pre-walk baseline.

Contrast ^a^	Change	Lower CI	Upper CI	*p*-Value	Likelihood (%) Decrease/Trivial/Increase	Qualitative ^b^
**mBF (mL·min^−1^·100 mL^−1^)**					**Resting mBF (mL·min^−1^·100 mL^−1^)**	
14 h–BL AqT–Control	0.0715	−0.0918	0.235	0.368	10.0/18.5/71.5	Unclear
38 h–BL AqT–Control	−0.0629	−0.171	0.0455	0.225	76.6/20.1/3.3	Likely decrease
62 h–BL AqT–Control	0.0413	−0.0372	0.120	0.269	2.75/32.1/65.2	Possible increase
					**10% Exercise mBF (mL·min^−1^·100 mL^−1^)**	
14 h–BL AqT–Control	−0.141	−0.536	0.253	0.436	35.0/62.5/2.5	Possibly trivial
38 h–BL AqT–Control	−0.154	−0.839	0.531	0.628	42.6/45.4/12.0	Unclear
62 h–BL AqT–Control	0.174	−0.529	0.876	0.593	11.3/43.5/45.2	Unclear
					**20% Exercise mBF (mL·min^−1^·100 mL^−1^)**	
14 h–BL AqT–Control	0.752	−1.24	2.75	0.407	6.52/34.9/58.6	Unclear
38 h–BL AqT–Control	0.104	−0.736	0.944	0.788	4.54/83.3/12.2	Likely trivial
62 h–BL AqT–Control	0.443	−0.703	1.59	0.409	2.81/56.5/40.7	Possibly trivial
**mVO_2_ (mLO_2_·min^−1^·100g^−1^)**					**Resting mVO_2_ (mLO_2_·min^−1^·100 g^−1^)**	
14 h–BL AqT–Control	−0.0155	−0.0382	0.0072	0.154	86.6/11.1/2.24	Likely decrease
38 h–BL AqT–Control	0.0052	−0.0030	0.0134	0.188	0.55/42.2/57.3	Possible increase
62 h–BL AqT–Control	−0.0051	−0.0135	0.0034	0.212	55.8/43.5/0.74	Possible decrease
					**10% Exercise mVO_2_ (mLO_2_·min^−1^·100 g^−1^)**	
14 h–BL AqT–Control	−0.0852	−0.238	0.0677	0.236	60.5/38.1/1.33	Possible decrease
38 h–BL AqT–Control	−0.0033	−0.104	0.0970	0.943	8.89/84.3/6.84	Unclear
62 h–BL AqT–Control	0.0310	−0.149	0.211	0.707	11.6/55.5/32.9	Unclear
					**20% Exercise mVO_2_ (mLO_2_·min^−1^·100 g^−1^)**	
14 h–BL AqT–Control	−0.0973	−0.291	0.0960	0.284	54.6/43.6/1.77	Possible decrease
38 h–BL AqT–Control	0.0508	−0.215	0.317	0.680	12.7/49.3/38.1	Unclear
62 h–BL AqT–Control	0.144	−0.229	0.516	0.411	8.5/28.4/63.0	Unclear

^a^ Data for each contrast are 14-h baseline, 38-h baseline and 62-h baseline. ^b^ The threshold for the smallest worthwhile change for a mechanism was 0.2 × SD [18]. The likelihood that a contrast was at least greater than the Cohen *d* threshold was: 25–75% possible; 75–95% likely; 95–99.5% very likely; >99.5% almost certain. Unclear refers to outcomes where the likelihood of both an increase and decrease exceeded 5% [18]. BL = baseline; AqT = Aqua Titan; 95% CI = confidence interval; mBF = muscle blood flow; mVO_2_ = muscle oxygen consumption; Hb = hemoglobin.

## Appendix A

### Appendix A.1. Near-Infrared Spectroscopy

A frequency-domain (FD) oximeter (Oxiplex TS, ISS Inc., Champaign, IL, USA) was used for NIRS data collection. The FD oximeter emitted light sources at two different wavelengths (four lasers at 690 nm and four at 830 nm) to measure concentrations of oxygenated hemoglobin [HbO_2_] and deoxygenated hemoglobin [HHb], which can be combined to give absolute total hemoglobin (t[Hb] = [HbO_2_] + [HHb]). The FD NIRS measures individual tissue’s optical properties, allowing for calculations of absolute hemoglobin measures. The eight light-emitting diodes were positioned on the oximeter at distances of 2.5, 3.0, 3.5 and 4.0 cm from the detector. The emitted light was modulated for light intensity, and the subsequent phase shift and amplitude were used to calculate µ_s_ and µ_a_ [34], using the OxiTS Software (ISS Inc., Champaign, IL, USA). The NIRS device was allowed to warm up for 30 min prior to each session, before a calibration was performed using a calibration block, and also checked against a block with known µ_s_ and µ_a_. At the end of each session, the NIRS probe was placed on the calibration block to confirm there had been no drift in µ_s_ or µ_a_.

### Appendix A.2. Muscle Microvascular Perfusion

Absolute local muscle perfusion was assessed as the average t[Hb] for a given period. Resting perfusion was calculated from the average of two t[Hb] selections taken after the participant was seated for at least 10 min. Perfusion during exercise was estimated by the average t[Hb] signal during the two-second rest period between plantar flexion movements when the foot was resting at 10° of plantar flexion.

### Appendix A.3. Muscle Microvascular Blood Flow

Hemoglobin was used as an endogenous intravenous tracer to determine mBF and mVO_2_ via VOs and AOs, respectively. A rapid-inflation cuff (Hokanson SC10D, Hokanson Inc., Bellvue, WA, USA) was placed as high as possible around the right thigh of the participant, to minimize levels of discomfort and any possible motion artefacts on the NIRS probe caused by tissue distortion during cuff inflation. The cuff was rapidly inflated using a custom-built pressure device in combination with a 40-L air compressor (Project Air, Spear & Jackson, Victoria, Australia) to a sub-systolic pressure (55–60 mmHg), subsequently preventing venous outflow, whilst arterial inflow was not affected [35]. Cardiac rhythm and heart rate were recorded through LabChart via a three-lead electrocardiogram setup, in conjunction with a custom-built trigger system to add markers at the start and end of occlusions. At rest, mBF was estimated over the first three cardiac cycles after the onset of occlusion. During exercise, only the first cardiac cycle after onset of occlusion was used to calculate mBF, due to the inclusion of additional cardiac cycles having been illustrated to underestimate mBF at higher arterial inflows (i.e., exercise) [36].

The slope of the t[Hb] signal during the VO was used to calculate mBF via conversion into units of mL per min, per 100 mL of blood (mBF (mL·min^−1^·100 mL^−1^) = $\left[\left(\frac{4}{\left[\mathrm{Hb}\right]}\cdot 100\right)\cdot \left(\frac{\Delta t\left[\mathrm{Hb}\right]}{\Delta t}\cdot 0.06\right)\right]$), where [Hb] is whole blood haemoglobin. Whole blood haemoglobin levels were measured each session via a finger-prick blood sample collected via Hb 201^+^ micro-cuvettes (HemoCue AB, Angelholm, Sweden) and analyzed in a Hb 201^+^ Analyzer (HemoCue AB, Angelholm, Sweden). The molecular mass of hemoglobin (64.458 g∙mol^−^^1^) and the ratio between hemoglobin and O_2_ molecules (1:4) were accounted for.

### Appendix A.4. Muscle Oxygen Consumption

Muscle oxygen consumption was estimated via the rate of change of the hemoglobin difference ([HbDif]) signal during an AO ([HbDif] = [HbO_2_] − [HHb]). The cuff was inflated to a supra-systolic pressure (280 mmHg) to inhibit venous outflow, as well as arterial inflow. Since t[Hb] is constant under occlusion, the resultant increase in [HHb] and subsequent decrease in [HbO_2_] reflects oxygen consumption as oxygen releases from the hemoglobin molecules and diffuses into the surrounding muscle. The [HHb] and [HbO_2_] signals were corrected for blood-volume changes and then used to calculate the [Hbdif] for an improved signal-to-noise ratio [37]. The slope of the [HbDif] signal was converted into milliliters of O_2_, per min, per 100 g of tissue (mVO_2_ (mLO_2_∙min^−1^∙100 g^−1^) = $abs\left[\frac{\Delta \left[HbDif/2\right]\cdot 60}{\left(10\cdot 1.04\right)\cdot 4}\right]\cdot \frac{22.4}{1000}$), assuming 22.4 L for the volume of gas (STPD) and 1.04 kg∙L^−1^ for muscle density [38].

**Table A1:** Statistical summary of the effect of Aqua Titan-treated stockings worn during the 62-h recovery period after an uphill walk on medial-lateral COP movement in a bilateral stance with eyes closed, relative to the pre-walk baseline.

Contrast ^a^	Change (Hq)	Lower CI	Upper CI	*p*-Value	Likelihood (%) Decrease/Trivial/Increase	Qualitative ^b^
					**Hq COPx (q = −3)**	
14 h–BL AqT–Control	−0.0003	−0.0527	0.0521	0.990	27.6/45.7/26.7	Unclear
38 h–BL AqT–Control	−0.0703	−0.124	−0.017	0.013	98.7/1.30/0.03	Very likely decrease
62 h–BL AqT–Control	0.0040	−0.0462	0.0543	0.865	21.1/46.5/32.3	Unclear
					**Hq COPx (q = 0)**	
14 h–BL AqT–Control	0.0078	−0.0331	0.0487	0.686	14.0/46.3/39.7	Unclear
38 h–BL AqT–Control	−0.0635	−0.109	−0.0184	0.009	99.2/0.82/0.02	Very likely decrease
62 h–BL AqT–Control	0.0054	−0.0417	0.0525	0.810	20.5/42.6/36.8	Unclear
					**Hq COPx (q = 5)**	
14 h–BL AqT–Control	−0.0097	−0.0439	0.0245	0.548	51.6/36.7/11.7	Unclear
38 h–BL AqT–Control	−0.0456	−0.0816	−0.0093	0.017	98.5/1.5/0.06	Very likely decrease
62 h–BL AqT–Control	0.0114	−0.0271	0.0498	0.550	13.7/31.7/54.6	Unclear

^a^ Data for each contrast are 14-h baseline, 38-h baseline and 62-h baseline. ^b^ The threshold for the smallest worthwhile change for the balance function of Hq was set at 0.2 × SD [18]. The likelihood that a contrast was at least greater than the Cohen *d* threshold was: 25–75% possible; 75–95% likely; 95–99.5% very likely; >99.5% almost certain. Unclear refers to outcomes where the likelihood of both an increase and decrease exceeded 5% [18]. BL = baseline; AqT = Aqua Titan; CI = 95% confidence interval.

**Table A2:** Statistical summary of the effect of Aqua Titan-treated stockings worn during the 62-h recovery period after an uphill walk on ankle joint range of motion, relative to the pre-walk baseline. ROM, range of motion.

Contrast ^a^	Change (°)	Lower CI	Upper CI	*p*-Value	Likelihood (%) Decrease/Trivial/Increase	Qualitative ^b^
					**Dorsiflexion ROM (°)**	
14 h–BL AqT–Control	0.657	−0.0607	1.38	0.070	0.00/81.6/14.4	Likely trivial
38 h–BL AqT–Control	1.09	−0.0783	1.03	0.065	0.01/44.6/55.4	Possible increase
62 h–BL AqT–Control	1.24	−0.226	2.70	0.091	0.08/37.2/62.8	Possible increase
					**Plantar Flexion ROM (°)**	
14 h–BL AqT–Control	0.630	−1.69	2.94	0.540	5.62/57.7/36.6	Possibly trivial
38 h–BL AqT–Control	1.26	0.326	2.21	0.013	0.00/28.8/71.2	Possible increase
62 h–BL AqT–Control	1.44	0.30	2.58	0.017	0.00/21.5/78.5	Likely increase
					**Total ROM (°)**	
14 h–BL AqT–Control	0.823	−0.833	2.48	0.302	0.46/70.8/28.8	Possibly trivial
38 h–BL AqT–Control	2.37	0.614	4.13	0.012	0.00/10.0/90.0	Likely increase
62 h–BL AqT–Control	2.70	0.974	4.42	0.005	0.00/4.54/95.5	Very likely increase

^a^ Data for each contrast are 14-h baseline, 38-h baseline and 62-h baseline. ^b^ The threshold for the smallest worthwhile change for ankle dorsiflexion, plantar flexion and total ROM was 0.2 × SD [18]. The likelihood that a contrast was at least greater than the Cohen *d* threshold was: 25–75% possible; 75–95% likely; 95–99.5% very likely; >99.5% almost certain [18]. BL = baseline; AqT = Aqua Titan; 95% CI = confidence interval.
